# Prevalence of most common human pathogenic *Campylobacter* spp. in dogs and cats in Styria, Austria

**DOI:** 10.1002/vms3.93

**Published:** 2018-01-22

**Authors:** Thomas Pölzler, Hans‐Peter Stüger, Heimo Lassnig

**Affiliations:** ^1^ Centre for Foodborne Infectious Diseases in Graz Department of Veterinary Microbiology (VEMI) Institute for Medical Microbiology and Hygiene, Graz (IMED Graz) Austrian Agency for Health and Food Safety (AGES) Graz Austria; ^2^ Division for Data, Statistics and Risk Assessment (DSR) Austrian Agency for Health and Food Safety Graz Austria

**Keywords:** *Campylobacter* species, *Campylobacter jejuni*, dogs, cats, prevalence

## Abstract

The aim of this study was to determine the frequency of occurrence of most common human pathogenic *Campylobacter* species, *Campylobacter jejuni (C. jejuni)* and *Campylobacter coli (C. coli)*, in dogs and cats in Styria, Austria. In the period from April 2010 to April 2012, 842 faecal samples from dogs and cats from Styria, Austria were examined for *Campylobacter (C.)* species (spp.). All samples were subjected to qualitative microbiological culture testing, and additionally, some of them have been studied using qualitative real‐time PCR. In microbiological culture, 5.9% of all samples investigated were *C. *spp. positive. With 3.1% out of positive samples, *C. jejuni* was the most common type. *Campylobacter upsaliensis (C. upsaliensis)* was detected only in 0.5% of the samples. The remaining positive samples (2.4%) were classified as *C. *species (sp.). *C. coli* could not be found in any of the samples. A higher prevalence of *C. jejuni* was found in kittens with 14.3% and in diarrhoeic dogs (7.4%) and cats (23.8%). The real‐time PCR revealed for dogs and cats together, 27% of *C. jejuni*‐positive faecal and 8% positive faecal swap samples. The obtained *C. jejuni* strains underwent antibiotic resistance testing using three different tests (agar diffusion, MIC testing and E‐test) with different numbers of antibiotics. From the antibiotics used in this study, several showed high test‐dependent resistance rates (cephalexin, cefovecin, kanamycin, sulfamethoxazole/trimethoprim, penicillin G, ciprofloxacin, enrofloxacin, marbofloxacin, nalidixic acid). Overall, the prevalence of *C. *spp. in this study was very low compared to others, with the exception of *C. jejuni* in kittens and diarrhoeic animals. The results of the real‐time PCR suggest that the rate of colonization of *C. jejuni* was actually higher than the results of the culture showed. As the resistance rates of *C. jejuni* isolates partly were very high, possible transmission of (multi‐) resistant *C. jejuni* strains to humans especially from kittens and diarrhoeic animals must be expected.

## Introduction

Infections with *Campylobacter (C.)* species (spp.) are still the leading causes of acute bacterial gastroenteritis in industrialized countries. Human Campylobacteriosis shows the highest incidence among bacterial notifiable diseases in Europe since 2005. In 2015, the overall incidence in the EU was 65.5 confirmed cases/100 000 inhabitants. The rate in Austria 2015 was 73 cases/100 000 inhabitants. In 2015, 229 213 confirmed cases of disease were reported in the EU (Anonymous 2007, 2016).

The most common clinical symptoms are diarrhoea, fever and abdominal pain. The infection is usually self‐limiting and subsides without treatment within 1 week. In some cases, however, postinfectious complications such as Guillain‐Barré syndrome and reactive arthritis may occur (Allos 1997; Nachamkin & Blaser 2000; Moore *et al*. 2005).

For infections, almost exclusively thermotolerant *C. *spp. are responsible. The predominant, disease‐causing germ is *Campylobacter jejuni (C. jejuni)* with at least 80–90% of cases. The second most common is with 10% *Campylobacter coli* (*C. coli)* (Nielsen *et al*. 1997; Gillespie *et al*. 2002).

The high incidence of *Campylobacter* infections in humans results in large costs for the public health system (Buzby *et al*. 1997; Mangen *et al*. 2004).

The main risk factors for *Campylobacter* infection include poultry meat and poultry meat products, contaminated drinking water or crops, raw or insufficiently heated milk and direct animal contact. Infections occur primarily due to poor kitchen hygiene when handling poultry meat and poultry meat products. Between 20 and 40% of all human, *Campylobacter* infections can be directly traced back to poultry meat and poultry meat products (Pebody *et al*. 1997; Nadeau *et al*. 2002; Vellinga & van Loock 2002) and 52–80% overall to sources which originate from the poultry sector (Mullner *et al*. 2009; Anonymous, 2010; van Gerve 2012).

In Austria, studies on the prevalence of *Campylobacter* spp. have been made primarily on livestock (Ziegler 1993a,b; Hein *et al*. 2003; Ursinitsch *et al*. 2005), with only one study on dogs (Balucinska 1995). Currently, small animals (dogs, cats) are considered as asymptomatic carriers of *C. *spp. However, there are also publications in which *C. *spp. was classified as primary or secondary pathogen which can trigger gastrointestinal symptoms in small animals (Fleming 1983; Burnens *et al*. 1992). A transfer from dogs and cats to humans or vice versa cannot be ruled out (Damborg *et al*. 2004). In particular, dog owners seem to have a significantly higher risk of infection with *C. jejuni* and *coli* from their pets (Mughini‐Gras *et al*. 2013). Isolation of *C. *spp. in small animals succeeds very often. The most commonly detected species were *Campylobacter upsaliensis* (*C. upsaliensis)* 39–98%, *C. jejuni* 1.2–51.2% and *C. coli* 0–9.8% (Hald *et al*. 2004; Koene *et al*. 2004; Keller *et al*. 2007; Gargiulo *et al*. 2008; Acke *et al*. 2006, 2009a,b; Parson *et al*. 2010; Salihu *et al*. 2010; Parsons *et al*. 2011; Badlik *et al*. 2014; Procter *et al*. 2014; Giacomelli *et al*. 2015; Holmberg *et al*. 2015; Olkkola *et al*. 2015; Selwet *et al*. 2015; Bojanić *et al*. 2017). However, the isolation rates from faeces differ greatly depending on age, clinical signs, environment, concomitant diseases, infections with other enteropathogenic organisms, respective *Campylobacter* species, isolation method and design of the study (Torre & Tello 1993; Engvall *et al*. 2003; Bender *et al*. 2005; Wieland *et al*. 2005; Chaban *et al*. 2010).

The primary objective of this study was to determine the prevalence of the most pathogenic thermotolerant *C. *spp. for humans (*C. jejuni*,* C. coli*) in faeces of dogs and cats from Styria, Austria. Additionally, it should be examined whether the age of the animals or gastrointestinal diseases have an influence on the occurrence of these *C. *spp.

As a supplement, antibiotic susceptibility testing of *C. jejuni* isolates was carried out.

## Materials and methods

### Sampling and shipment

In the period between April 2010 and April 2012, 842 samples (498 dogs, 344 cats) were examined for *Campylobacter (C.)* species (spp.). They consisted of 756 faecal swabs (442 dogs, 314 cats) and 70 faecal samples (51 dogs, 19 cats) (Table 1), which were gathered from the rectum of the animals by practicing veterinarians from Styria during routine investigations using sterile cotton swab and placed in a hermetically sealed tube containing nutrient medium (Amies W, Switzerland; Sterilin Ltd., Newport Gwent, UK). The extracted faecal samples containing at least 1 g of faeces were placed in sterile stool tubes with spoon (76 × 20 mm; Sarstedt, Germany), sealed and protected by a screw top vessel (85 × 30 mm; Sarstedt, Germany).

**Table 1:** *Campylobacter* species‐positive dogs and cats (all samples)

Animal species	*C. jejuni*		*C. coli*		*C. upsaliensis*		*C*. species		Total negative		Total positive		Total samples	
	*n*	%	*n*	%	*n*	%	*n*	%	*n*	%	*n*	%	*n*	%
Dogs	11	2.2	0	0	4	0.8	13	2.6	470	94.3	28	5.6	498	59.1
Cats	15	4.4	0	0	0	0	7	2.0	322	93.6	22	6.4	344	40.9
Total	26	3.1	0	0	4	0.5	20	2.4	792	94.1	50	5.9	842	100

Until shipping, the samples were refrigerated at a temperature from 2 to 8°C. A maximum of 4 days was set between sampling and arrival at the institute. The samples were shipped together with a submission form by a messenger in an uncooled padded envelope.

Moreover, 16 faecal samples which were obtained from autopsies in our own institute were examined on the day of removal.

The samples were divided into two groups based on history, one group of animals suffering from gastrointestinal symptoms and another group not suffering this symptoms (healthy or having other diseases).

### Qualitative microbiological culture method (ISO 10272‐1/2006)

For sample collection and selected microbiological culture, simple and rapid methods (faecal swabs, direct plating on mCCDA agar) were chosen, to keep effort and costs low and to be able to examine as many samples as possible. In literature, there was no clear indication which sample material or which cultural detection method would be the best of all. Only the combination of several methods seems to provide best results (Koene *et al*. 2004; Acke *et al*. 2006, 2009a,b).

After arrival at the institute, the samples were stored until further processing at refrigerator temperature (2–8°C). Further processing of the samples was carried out on the same day. First, the required selective agar plates (mCCDA agar) were brought to room temperature. The faecal swabs were streaked directly and faecal samples with a laboratory wire loop on the mCCDA agar. Then, two dilutional streaks with an annealed laboratory wire loop were made. The plates were incubated for 48 h at 37°C under microaerophilic conditions (CampyGen CN0035A, 3.5 L; Oxoid, Hampshire, UK) either in a glove box (Scholzen Microbiology Systems AG, Switzerland MC 1G) or an anaerobic box (AnaeroPack Rectangular Jar, 2.5/7 L; Mitsubishi Gas Chemical Company Inc., Japan). A portion of the faecal swabs (*n* = 84) was placed after the smear in a Preston enrichment broth (PEB) and was incubated for 24 h at 37°C under microaerophilic conditions. The faecal samples were either immediately DNA extracted or frozen at −20°C until processing.

Three drops (100 *μ*L) of the enrichment broth were pipetted on a selective agar plate mCCDA (Campylobacter blood‐free selective agar base, CM0739; Oxoid/UK, CCDA selective supplement, SR0155; Oxoid) and CASA (Campylobacter Selective Agar 20 BT 90; Chemunex AES, France), two dilutional streaks with an annealed laboratory wire loop were made and the plates were incubated under microaerophilic conditions at 37°C for 48 h. In addition, from some of the samples (*n* = 75), 1 mL supernatant of the enrichment broth was taken out and transferred into a 1.5 mL Eppendorf tube for DNA extraction. These samples were frozen at −20°C till processing. DNA extraction was performed using QIAamp DNA Stool Mini Kit (Qiagen, Hilden, Germany) according to the manufacturer's instructions. The plates were checked after 48 h, suspect *Campylobacter* colonies subcultured on sheep blood agar COS (Biomérieux, France) and incubated under microaerophilic conditions at 37°C for 24–48 h. Differentiation was performed by Gram staining, phase contrast microscopy, oxidase (oxidase test strip, product no. 1.13300.0001; Merck, Germany), catalase (catalase colour ID, product no. 55561, Biomérieux), hippuric acid‐ (hippuric sodium salt, Product No. 820648.0025; ninhydrin, Product No. 106762.0010; VWR, Radnor, Pennsylvania, USA) and indoxyl acetate reaction (indolyl acetate, synonym:.. indoxyl acetate, Product No. 820706.0001; VWR). Hippuric positive strains were identified as *C. jejuni* strains and preserved in liquid nitrogen. Hippuric acid negative and indoxyl acetate positive respectively double negative strains were conserved in liquid nitrogen as well as boiled and frozen at −20°C for DNA real‐time PCR analysis.

### Qualitative real‐time PCR (LaGier *et al*. 2004)

In this method, the hippuricase gene served as target gene, which only occurs in *C. jejuni*. The *C. jejuni* strain typing using real‐time PCR is more reliable than with the biochemical Hippuric acid reaction, because hippuricase negative strains (≤10%), in which there is no expression of the hippuricase gene, will also be detected.

Real‐time PCR approach (20 *μ*L): 8.4 *μ*L H2O/PCR grade, 2 *μ*L LightCycler FastStart DNA Master HybProbe Mix (Roche Diagnostics, Rotkreuz, Switzerland), 1.6 *μ*L MgCl2 (25 mM), 1 *μ*L primers and probe (500 nM; Metabion, Planegg/Steinkirchen, Germany) and 5 *μ*L supernatant of the sample. The amplification reaction was run according to the following programme on a LightCycler 2.0 (Roche Diagnostics, Rotkreuz, Switzerland) From: 1 cycle of 95°C for 10 min; 50 cycles each at 95°C for 15 s, 60°C for 1 min. For the positive and negative control reference, strains of *C. jejuni* DSMZ 4688 and *C. coli* DSMZ 4689 were used.

The remaining hippuricase negative respectively double negative strains were further differentiated by PCR (Wang *et al*. 2002; Jensen *et al*. 2005).

### Antibiotic sensitivity testing

Antibiotic sensitivity testing was performed on 13 *C. jejuni* strains. For this, three different tests were used with various antibiotics.

#### Agar diffusion test (AGES/IVET Graz)

The bacterial strain to be tested was streaked out on a Mueller‐Hinton agar plate with 5% sheep blood (Biomérieux). After that, antibiotic test plates with a defined concentration were applied on the agar plates and incubated under microaerophilic conditions 48 ± 2 h at 37 ± 1°C. The inhibition zones were measured and evaluated according to CLSI (Clinical and Laboratory Standards Institute) as sensitive, intermediate or resistant.

The antibiotics chosen for this test are routinely and frequently used in small animal practice (amoxycillin, ampicillin, cephalexin, chloramphenicol, enrofloxacin, gentamycin, kanamycin lincospectin, marbofloxacin, neomycin, penicillin G, streptomycin, tetracycline, sulfamethoxazole/trimethoprim, cefovecin).

#### MIC testing (AGES/IMED Graz)

For the implementation of the MIC testing, the strains to be tested were plated out on Columbia blood agar (Biomérieux) and incubated under microaerophilic conditions 44 ± 4 h at 37 ± 1°C. This culture was used to prepare a suspension according to McFarland 0.5. From this suspension, 50 *μ*L was transferred in Mueller‐Hinton broth with TES/lysed Horse Blood (TREK Diagnostic Systems; Thermo Scientific, Waltham, MA, USA). The resistance determination was performed using the Sensititre^®^ system (TREK Diagnostic Systems; Thermo Scientific), a technique for determining the MIC value. For this purpose, dehydrated AB gradients were applied to the wells of a microtiter plate, with the appropriately prepared bacterial suspension inoculated (with automatically inoculator) and incubated 44 ± 4 h at 37 ± 1°C under microaerophilic conditions. For the evaluation of the microtiter plates, SensiTouch^®^ system was used. In this system, step by step, each AB gradient was accessed. The following antibiotics were used: ampicillin, amoxycillin/clavulanic acid, chloramphenicol, ciprofloxacin, colistin, erythromycin, gentamycin, imipenem, nalidixic acid, neomycin, streptomycin, tetracycline.

#### E‐test (AGES/IVET Graz)

The E‐test (Biomérieux) is a quantitative method for the determination of antibiotic susceptibility of bacteria. An Epsilon test strips, which was coated with a defined, ascending concentration of an antibiotic, was placed on Mueller‐Hinton agar with 5% sheep blood (Biomérieux) after plating out the bacterial strain. Then, the plate was incubated for 48 ± 2 h at 37°C under microaerophilic conditions, the MIC value (point of intersection between the E‐test strips and the germ growth boundary) was read. This test was only used for enrofloxacin.

### Statistical methods

For statistical analysis of associations, the free software r (3.4.0) was used. To check for significant associations, an exact Fisher test was performed.

## Results

A total of 50 samples (5.9%; *n* = 842) were tested positive for *C*. spp. (Table 1). Among these were 26 (3.1%) *C. jejuni* and 4 (0.5%) *C. upsaliensis*‐positive samples (Table 1). The remaining 20 samples could not be assigned to the most important human‐relevant species (*C. jejuni, C. coli, C. lari, C. fetus ssp., C. upsaliensis*) and were therefore not further differentiated.

The number of positive samples in dogs was lower (5.6%; *n* = 28), measured by the total number of samples of this species, compared with cats 22 (6.4%). The number of positive *C. jejuni* samples in dogs (*n* = 11, 2.2%) was less than in cats (*n* = 15, 4.4%) (Table 1). These differences were not statistically significant (α = 0.05). No statistically significant differences in regard to any species were found.

In young dogs (<1 year; *n* = 27), no sample could be tested positive for *C. jejuni*. Among the adult dogs (>1 year; *n* = 450) 11 (2.4%), *C. jejuni*‐positive samples were found. This difference was not statistically significant (α = 0.05).

In contrast, in juvenile cats (<1 year; *n* = 28) 4 (14.3%), *C. jejuni*‐positive animals were detected. In comparison, in adult cats (>1 year; *n* = 288) 11 (3.8%), *C. jejuni*‐positive were observed (Table 2). For this evaluation, only those individuals with exact information of age in the anamnesis were used. This result was statistically significant (*P* = 0.034). In the groups with accurate medical history regarding diarrhoea (dogs: *n* = 135; cats: *n* = 118), the number of *C. jejuni*‐positive animals in dogs was 2 (7.4%; *n* = 27) and in cats 5 (23.8%; *n* = 21). Two healthy dogs (1.9%; *n* = 108) and three healthy cats 3 (3.1%; *n* = 97) were *C. jejuni* positive (Table 3). For the remaining animals which were examined, no information on health status was provided.

**Table 2:** *Campylobacter* species‐positive dogs and cats: Ratio between young animals and adults (only samples with declaration of age)

Animal species	Age	*C. jejuni*		*C. coli*		*C. upsaliensis*		*C*. species		Total negative		Total
		*n*	%	*n*	%	*n*	%	*n*	%	*n*	%	*n*
Dogs	<1 year	0	0	0	0	0	0	1	3.7	26	96.3	27
	>1 year	11	2.4	0	0	4	0.9	11	2.6	424	94.1	450
Cats	<1 year	4	14.3	0	0	0	0	0	0	24	85.7	28
	>1 year	11	3.8	0	0	0	0	7	2.4	270	93.8	288

**Table 3:** *Campylobacter* species‐positive dogs and cats: Ratio between healthy and diarrhoeic animals (only samples with accurate health information)

	*C. jejuni*		*C. coli*		*C. upsaliensis*		*C*. species		Total negative		Total
	*n*	%	*n*	%	*n*	%	*n*	%	*n*	%	*n*
Dogs											
Healthy	2	1.9	0	0	0	0	2	1.9	104	96.2	108
Sick	2	7.4	0	0	0	0	1	3.7	24	88.9	27
Cats											
Healthy	3	3.1	0	0	0	0	1	1	93	95.9	97
Sick	5	23.8	0	0	0	0	0	0	16	76.2	21

The faecal samples which were examined by real‐time PCR showed 23% (*n* = 15) and the faecal swab enrichments 8% (*n* = 75) positivity for *C. jejuni*.

A comparison between mCCDA‐ and CASA agar (*n* = 86) revealed 2.3% *C. *spp.‐positive samples on both agars at direct plating of faeces. When comparing direct plating on mCCDA‐ versus enrichment in Preston broth and striking out on mCCDA agar (*n* = 84), 9.5% and 6% were found positive, respectively.

**Figure 1:**
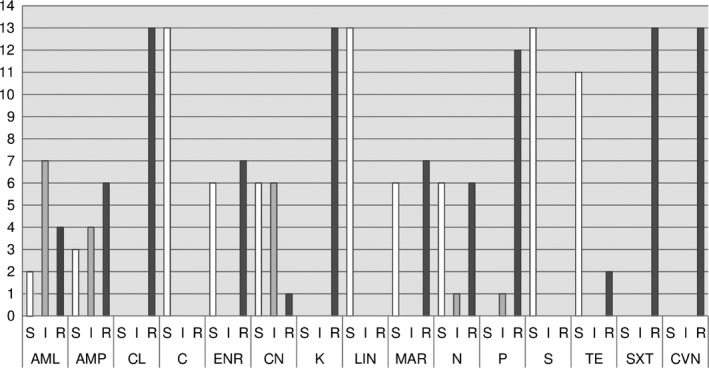
Antibiotics – agar diffusion test.

**Figure 2:**
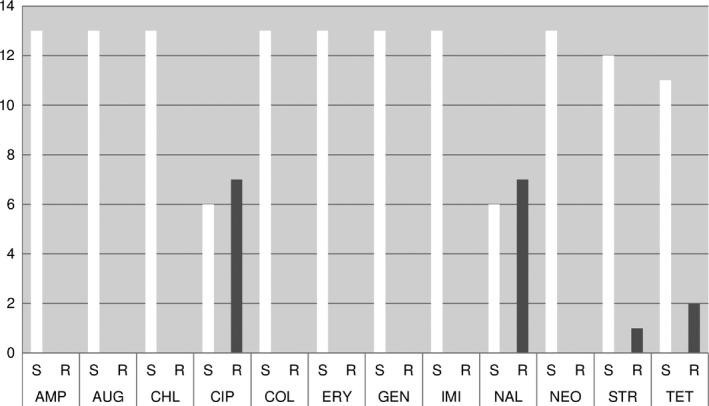
MIC – testing (CLSI – clinical breakpoints).

**Figure 3:**
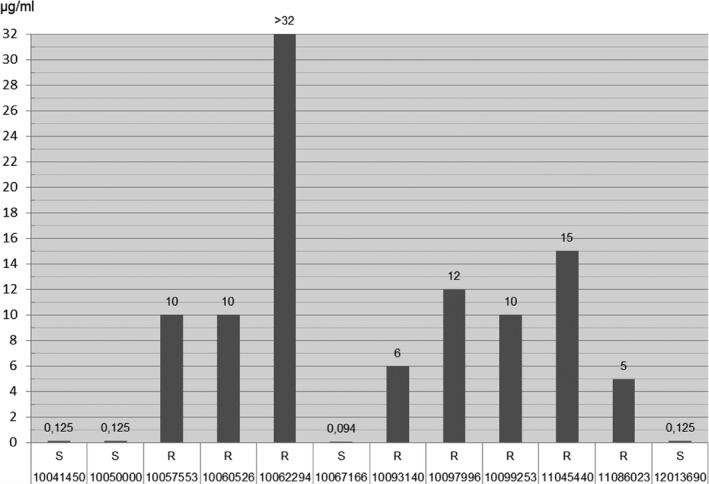
Etest ^®^: Enrofloxacin.

The results of the antibiotic susceptibility testing of 13 *C. jejuni* strains are shown in Figs 1, 2, 3.

## Discussion

In our studies, we could not confirm the reported high detection rates of *C. *spp. as mentioned in the literature (Balucinska 1995; Koene *et al*. 2004; Acke *et al*. 2006, 2009a,b,c; Gargiulo *et al*. 2008; Parsons *et al*. 2011; Badlik *et al*. 2014; Procter *et al*. 2014; Olkkola *et al*. 2015; Selwet *et al*. 2015). Possible reasons for this difference could be our focus on the most common human pathogenic species *C. jejuni* and *C. coli* which led to a study design not ideal for the detection of *Campylobacter upsaliensis*. Further reasons are the lower sensitivity of rectal swabs, in some cases a delay in plating out and the time allowed for *in vitro* growth.

The most frequently detected *Campylobacter* species was *C*. *jejuni*, the second most common type was *C*. *upsaliensis*. These results stand in contrast to most of the above‐mentioned publications. The isolation rate of *C. *spp. in faeces of dogs and cats differed primarily in relation to the age of the animals (Torre & Tello 1993; Engvall *et al*. 2003; Hald *et al*. 2004; Bender *et al*. 2005; Wieland *et al*. 2005; Acke *et al*. 2009a,b,c; Holmberg *et al*. 2015), the predominant *Campylobacter* species (Hald *et al*. 2004; Wieland *et al*. 2005; Acke *et al*. 2009a,b,c) and the isolation method used (Koene *et al*. 2004; Acke *et al*. 2006, 2009a,b,c).

Testing for the presence of *C. jejuni* with real‐time PCR, 27% positive faecal samples and 8% positive faecal swabs were found. This suggests that the colonization rate was actually higher. Chaban *et al*. (2010) found 7% *C. jejuni*‐positive samples in healthy and 46% positives in diarrhoeic dogs.

In dogs, no noticeable age‐dependent differences in *Campylobacter* positives between young animals under 1 year and adults were found. However, not a single specific *C. *spp. could be found in young dogs. Among the adult dogs, the amount of *C. jejuni* and *C. upsaliensis*‐positive samples was also very low. Selwet *et al*. (2015) found more *Campylobacter* spp. positive in young dogs (60%) than in adults (38.9%). Hald *et al*. (2004) have created an accurate excretion pattern for thermotolerant *C. *spp. and found that the rate of *Campylobacter* carriers in dogs during development increases from 60% in young animals of 3 months to nearly 100% in animals of 12 months and falls back to 67% at the age of 24 months.

Balucinska (1995)identified 45.3% *C. *spp.‐positive animals among young dogs and only 26.6% *C. *spp. positives among adult dogs in Austria. In diarrhoeic dogs, the number of *C. *spp. as well as *C. jejuni*‐positive animals was higher than the total positives.

Very striking was the difference in juvenile cats below 1 year of age compared to the total number of *C. *spp.‐positive cats, while the percentage of *C. *spp.‐positive adult cats was well within the range of the total positives. It should be emphasized that there were without exception only *C. jejuni*‐positive animals among positive kittens. This result was statistically significant (*P* = 0.034). Bender *et al*. (2005) found 30% *Campylobacter*‐positive among juveniles but only 3% positive cats among adults. Gargiulo *et al*. (2008) revealed in his studies of stray cats 27.7% *C. jejuni*‐positive adult and only 2.1% positive juvenile cats.

The group of diarrhoeic cats had the highest proportion of *C. *spp positives. All positive diarrhoeic cats were positive for *C. jejuni*.

Acke *et al*. (2006, 2009b) noticed in dogs and cats with diarrhoea, especially in those under 6 months, high prevalence of *C. *spp. The differences between diseased and healthy animals as well as between the age groups were largely insignificant. In the animals with diarrhoea, *C. jejuni* was the most abundant species (Acke *et al*. 2009b).

Balucinska (1995) found 31.6% *C. *spp. samples positive on diarrhoeic and 29.9% in healthy dogs in Austria.

These results suggest a certain zoonotic potential in diarrhoeic and/or juvenile dogs and/or cats. In the AB‐susceptibility testing of *C. jejuni* strains using agar diffusion test, MIC testing and E‐test high to medium resistance rates for enrofloxacin (ENR), ciprofloxacin (CIP), nalidixic acid (NAL), marbofloxacin (MAR), ampicillin (AMP), amoxycillin (AML) and tetracycline (TE) were found in descending order of frequency. According to Balucinska (1995), the rate of resistance for ciprofloxacin and enrofloxacin was 3% in dogs in Austria. In Ireland, Acke *et al*. (2009c) found with E‐test the following rates of resistance: 37.3% nalidixic acid, 19.6% ciprofloxacin, 13.7% tetracycline, 13.7% ampicillin, 11.8% erythromycin (ERY). In Poland, Andrzejewska *et al*. (2013) found the following rates of resistance: 64% ciprofloxacin, 16% tetracycline and 9% erythromycin. In comparison, the resistance rates of *C. jejuni* isolates from human, food (poultry) and primary production (poultry) samples were at 65.4%/53.6%/69.0%, nalidixic acid 64.4%/50.0%/60.3%, tetracycline 31.0%/23.8%/17.2%, erythromycin 0.3%/0.0%/0.0%, ampicillin 28.0%/22.6%/not performed (Anonymous, 2011).

In this study, when comparing the methods, matching results were mainly found in gyrase inhibitors (cipro‐, enro‐, marbofloxacin, nalidixic acid) and tetracyclines. The high rates of resistance in gyrase inhibitors and tetracyclines are comparable to those of the above‐mentioned recent literature. This indicates a certain transmission risk of (multi) resistant, potentially human pathogenic *C. jejuni* strains from dogs and cats to humans.

## Source of funding

This study was funded by Austrian Agency for Health and Food Safety (AGES).

## Conflict of interest

The authors declare that they have no conflict of interest.

## Ethics statement

The authors confirm that the ethical policies of the journal, as noted on the journal's author guidelines page, have been adhered to. No ethical approval was required because no experimental animals were used.

## Contributions

TP contributed to the designing of the study, did the data collection and writing of the draft manuscript, the final manuscript write up and editing, the writing of the final version of the manuscript and coordinated the publication of this manuscript. HPS did the statistical analysis. HL did the designing of the study, the proof reading of the manuscript and the supervision of the project. All authors read and approved the final manuscript.
